# A new approach to surgical correction of double-outlet right ventricle with remote interventricular communication

**DOI:** 10.1016/j.xjtc.2024.03.006

**Published:** 2024-03-16

**Authors:** Hamood Al Kindi, Madan Mohan Maddali, Pranav Kandachar, Faiza Al Kindi, Abdullah Al Farqani, Justin T. Tretter, Robert H. Anderson

**Affiliations:** a Cardithoracic Unit, Department of Surgery, Sultan Qaboos University Hospital, Seeb, Oman; b Department of Cardiothoracic Surgery, National Heart Center, The Royal Hospital, Muscat, Oman; c Department of Cardiac Anesthesia, National Heart Center, The Royal Hospital, Muscat, Oman; d Department of Cardiac Imaging, National Heart Center, The Royal Hospital, Muscat, Oman; e Department of Pediatric Cardiology, National Heart Center, The Royal Hospital, Muscat, Oman; f Cardiovascular Medicine Department, Heart, Vascular, and Thoracic Institute, Cleveland Clinic Foundation, Cleveland, Ohio; g Biosciences Institute, Newcastle University, Newcastle upon Tyne, United Kingdom

**Figure:**
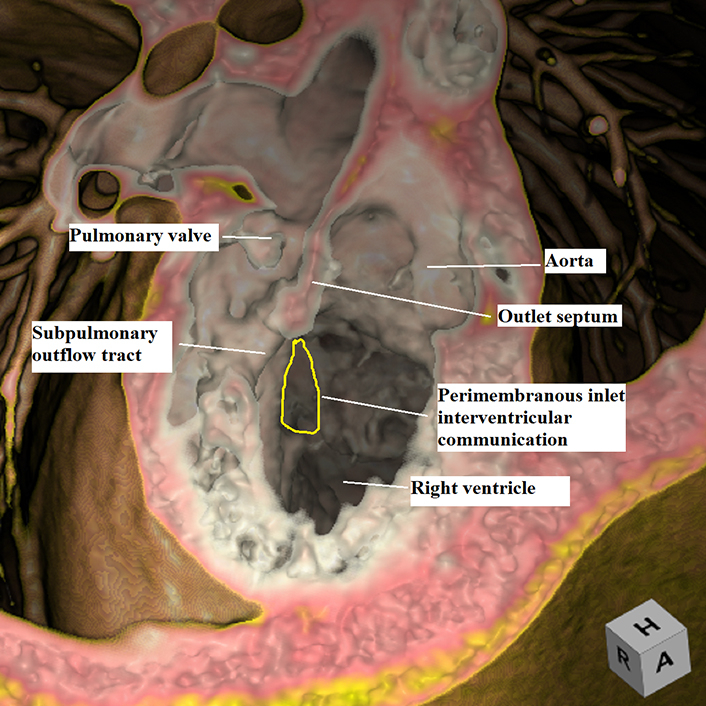
Virtual dissection showing the remoteness of the inlet interventricular communication.

**Central Message**

Challenges remain when correcting double-outlet right ventricle with a remote interventricular communication. We created a new communication, with excellent hemodynamic results.

Significant challenges are faced by surgeon to achieve biventricular repair when, in the setting of double-outlet right ventricle, the interventricular communication is remote from the arterial roots. This is known to occur in up to one-fifth of patients.^1^ Having recently encountered a patient in this setting, we were able to construct an intraventricular baffle redirecting the left ventricle to the aortic root, having created a new outlet alongside the existing uncommitted communication. The parents of the patient provided informed written consent for the publication of these data (February 3, 2024; Institutional Review Board No.: Moh/CSR/CR/23/40).

## Case Report

A 1-year-old boy, known to have complex congenital cardiac anomalies, was referred to our center for evaluation. Transthoracic echocardiography (Video 1) revealed a mirror-imaged heart with double-outlet right ventricle and bilateral infundibulums. The aortic root was anterior and left-sided relative to the pulmonary root. There was severe pulmonary valvular stenosis, with dysplastic leaflets and subvalvular obstruction. The interventricular communication was perimembranous and opened to the inlet of the right ventricle, remote from the arterial roots.

**Figure 1:**
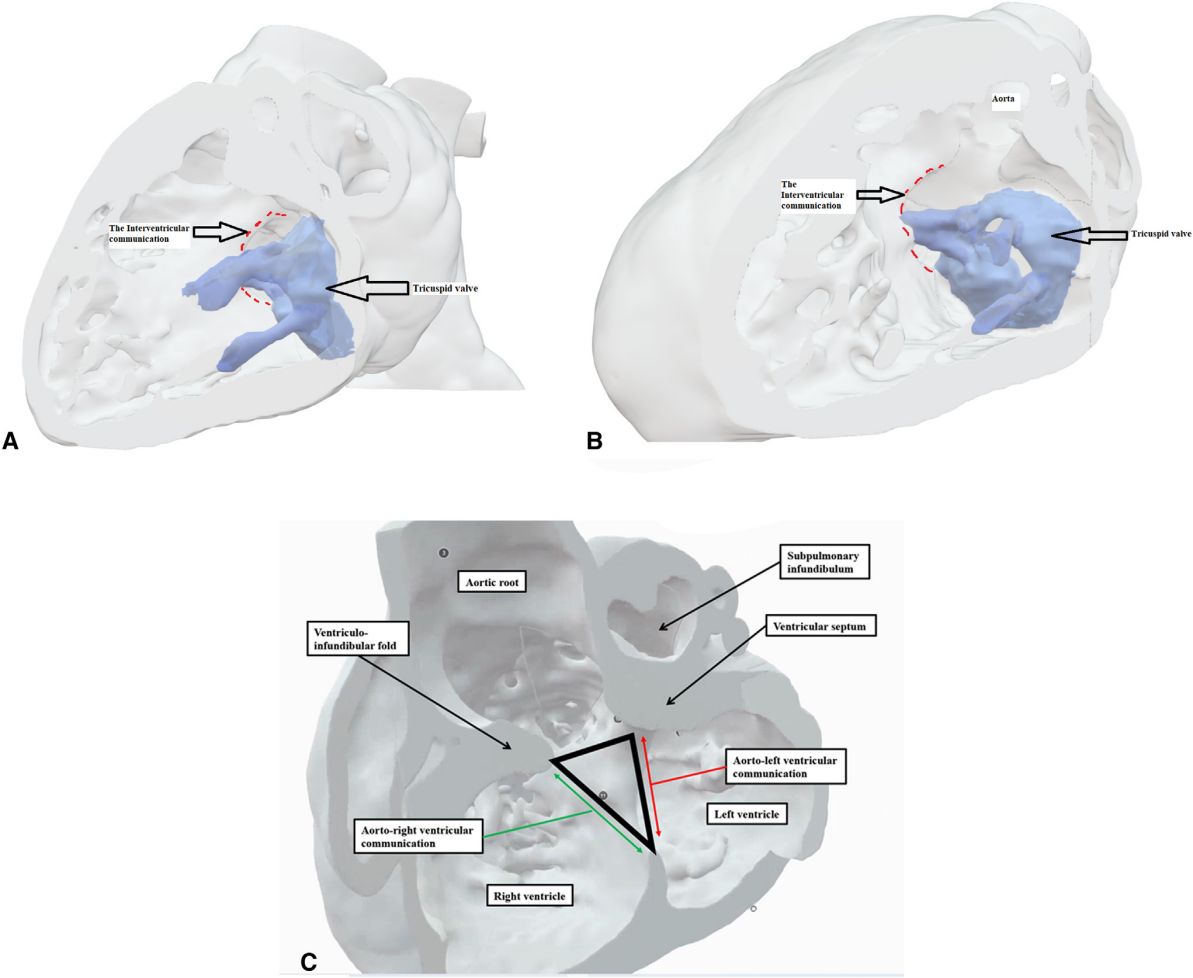

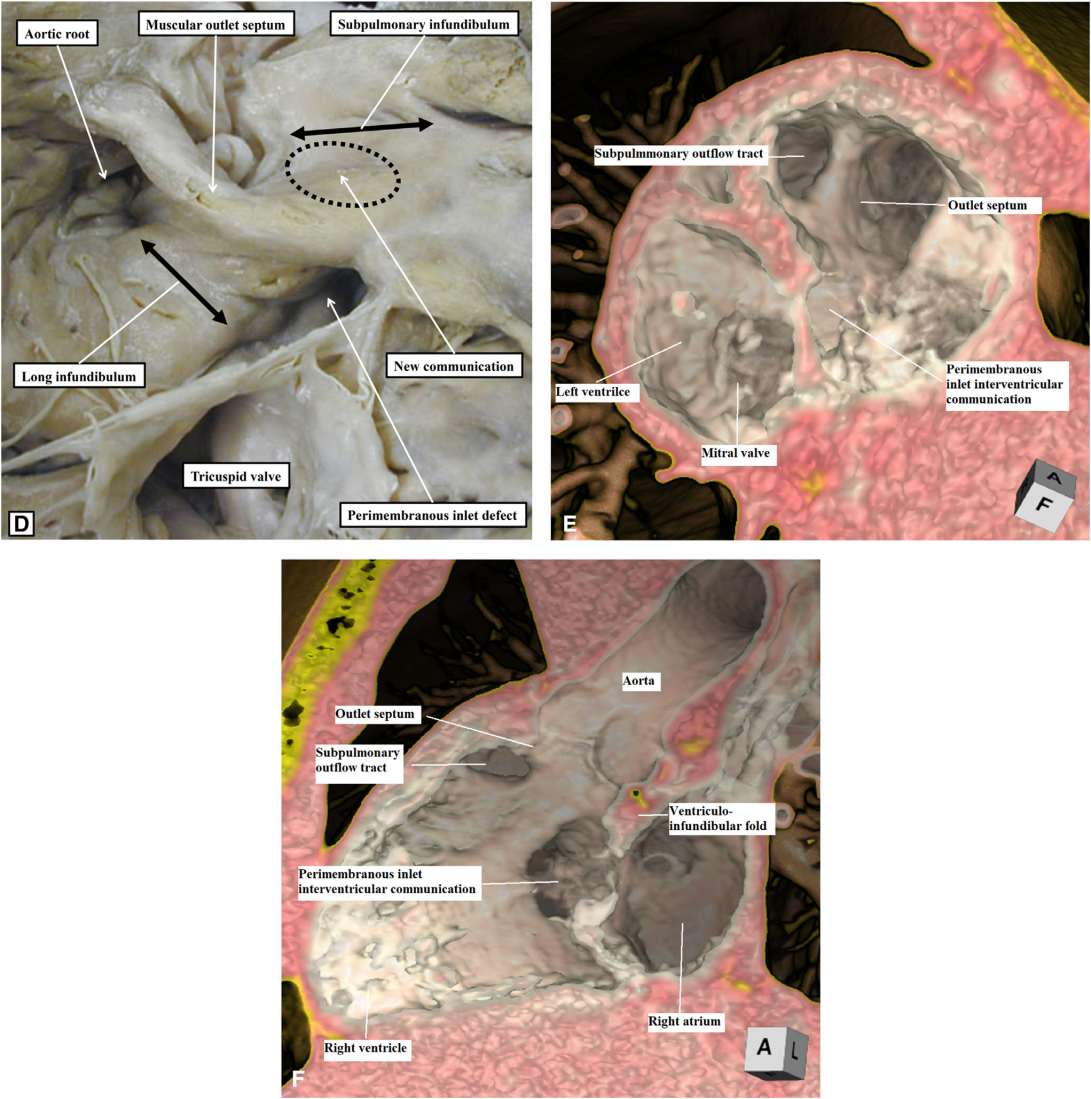
A and B, Three-dimensional modeling, showing the view from the right ventricle (A and B), reveals the location of the perimembranous interventricular communication in relation to the orifice of the tricuspid valve and the aortic root. C, The coronal view shows the interventricular communication, despite its remoteness, to be directly below the aortic root. D, A comparable morphological specimen. The defect again opens to the inlet of the right ventricle, yet is directly below the aortic root. We have previously described this defect as being subaortic. We now recognize that, as in the current case, it is remote, albeit still potentially related to the aorta. The oval shows the site of creation of the new communication. E and F, Virtual dissections of the 3-dimensional dataset show the internal anatomy as viewed from the *left* and *right sides* of the ventricular septum.

**Figure 2:**
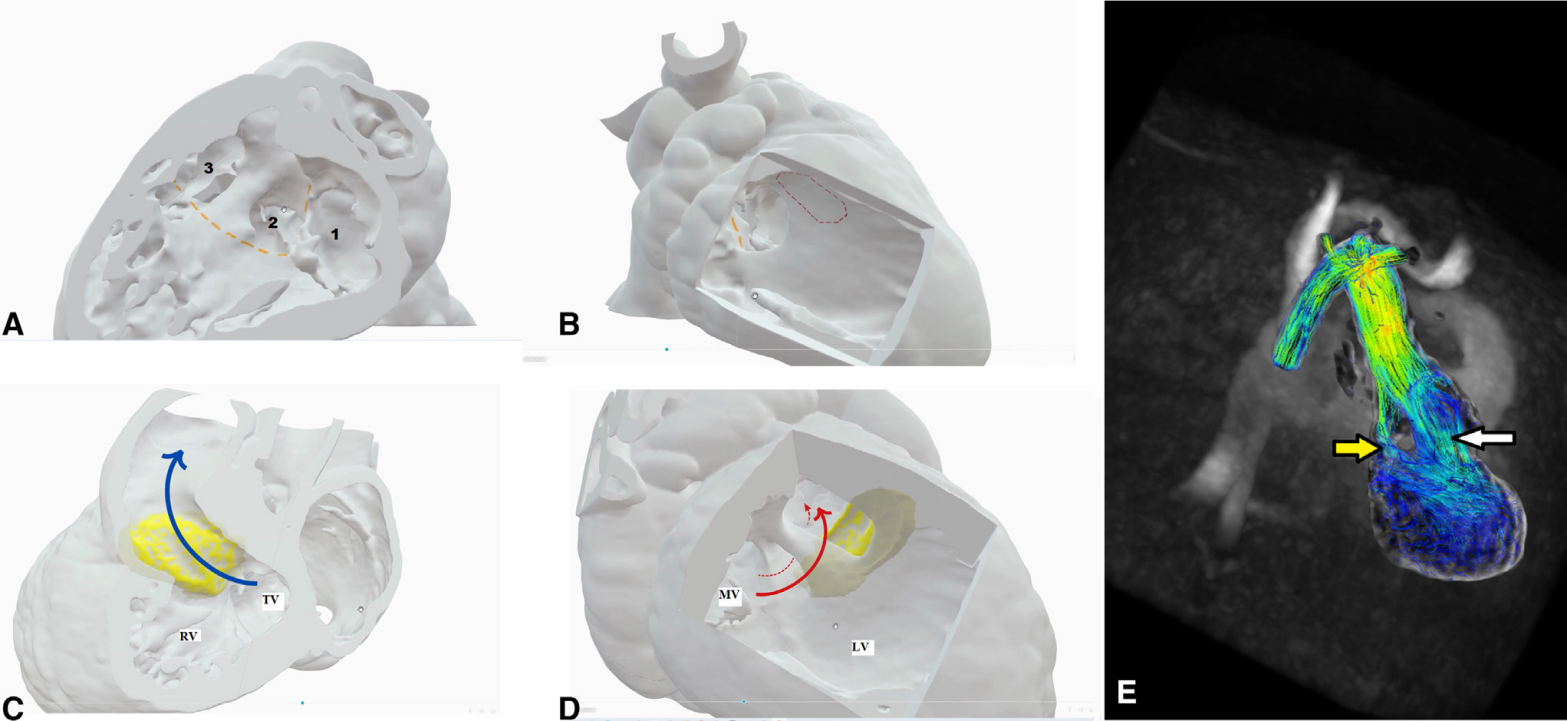
Preoperative modeling permitted us to anticipate the site of a new interventricular communication, which would open to the subpulmonary outlet tract. The panels show its site as seen from the *right* (A) and *left* (B) ventricles, along with the planned suture line (*yellow lines*), where 1 indicates the tricuspid valve, 2 indicates the original interventricular communication, and 3 indicates the new interventricular communication (marked in *red* in B). Postoperative modeling shows the subsequent anatomy and the flow from both interventricular communications to the aorta, again as seen from the *right* (C) and the *left* (D) ventricles. E, Magnetic resonance imaging permitting 4-dimensional analysis of flow. Of the flow, three-quarters was through the new interventricular communication (*white arrow*), rather than the original defect (*yellow arrow*). *RV*, Right ventricle; *TV*, tricuspid valve; *MV*, mitral valve; *LV*, left ventricle.

To aid in surgical planning, we made a contrast computed tomography scan (Video 1), followed by modeling and printing (Figure 1, *A-C*, and Video 1) of the 3-dimensional (3D) dataset. Intraoperative evaluation confirmed the location of the interventricular communication. It was remote from the aorta, with the leaflets and cords of the tricuspid valve covering its upper margin. We noted the potential to create a new interventricular communication in the subpulmonary outflow tract (Figure 2, *A* and *B*, and Video 1). Having created the new outlet, we placed a baffle in the right ventricle to direct both communications into the aortic root. Confirming the right ventricle to be of adequate size, we then placed a 16-mm Contegra conduit (Medtronic) to the pulmonary arteries. Although the patient had moderate tricuspid regurgitation, with diastolic right ventricle dysfunction, this improved with medical management such that the patient could be discharged from hospital on the 14th postoperative day.

Subsequent postoperative evaluation after 2 months, including additional 3D modeling (Figure 2, *C* and *D*, and Video 1), revealed an unobstructed pathway from the left ventricle to the aorta. A 4-dimensional magnetic resonance imaging scan demonstrated that three-quarters of flow from the left ventricle was across the newly created interventricular communication (Figure 2, *E*).

## Comment

**Figure 3:**
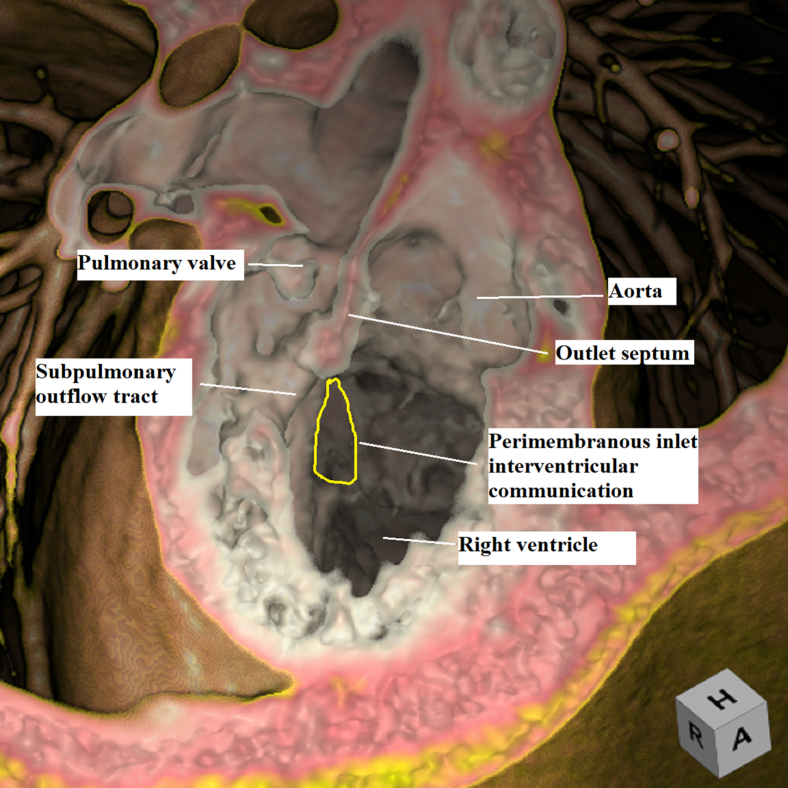
Virtual dissection showing the remoteness of the inlet interventricular communication.

The relationship of the interventricular communication, when both arterial trunks arise exclusively from the right ventricle, depends on its location within the right ventricle relative to the arterial roots, and the length of the infundibulums.^2^ In this setting, it can be difficult to determine the precise anatomy using cross-sectional echocardiography. The use of 3D modeling and printing has proven its value in surgical planning.^3^ Virtual dissection of the volume-rendered 3D computed tomography datasets also permits visualization of the intracardiac anatomy in a contextually appropriate manner.^4^ Such virtual dissection in our patient (Figure 1, *E* and *F*, and Figure 3) showed a defect that opened to the inlet of the right ventricle inferior to the caudal limb of the septomarginal trabeculation. The presence of tricuspid-to-mitral fibrous continuity confirmed its perimembranous nature, permitting us to infer that the conduction axis would be located posteroinferiorly.^5^ Despite the remote nature of the defect, it was aligned with the subaortic outflow tract. To commit the defect to the aorta would have required its extensive enlargement, along with detachment of some of the cords supporting the leaflets of the tricuspid valve. We deemed that such an approach might have mandated the use multiple patches,^1^^,^^E1^^,^^E2^ with the potential risk of reinterventions on the tricuspid valve, heart block, and obstruction of the outlet from the left ventricle. Enlarging the defect toward the subpulmonary outflow tract and adding an arterial switch^E3^ was deemed inappropriate due to the stenosis of the pulmonary valve. We decided, therefore, to create an additional defect opening from the left ventricle to the subpulmonary infundibulum, having shown the right ventricle to be sufficiently large to accommodate a baffle connecting both arterial roots to the left ventricle while still permitting construction of a right ventricle-to-pulmonary arterial conduit.

The need to create or enlarge an interventricular communication arises when the defect is the outlet for the morphologic left ventricle.^E4^ To our knowledge, ours is the first deliberate attempt to create an additional communication as an alternative surgical approach when correcting double-outlet right ventricle with a remote interventricular communication. Our postoperative scan using 4-dimensional magnetic resonance imaging showed that the maneuver created a more direct route to the aorta compared with the original inlet perimembranous defect. Theoretically, it would have been plausible simply to close the original interventricular communication, retaining only the new communication as the left ventricular outlet. This approach may be considered in future cases, although we would be wary that the new defect, having exclusively myocardial borders, might reduce spontaneously in size.

## Conflict of Interest Statement

The authors reported no conflicts of interest.

The *Journal* policy requires editors and reviewers to disclose conflicts of interest and to decline handling manuscripts for which they may have a conflict of interest. The editors and reviewers of this article have no conflicts of interest.

## Supplementary Data

Video 1 The video clip, with its commentary, summarizes the case report and the 3-dimensional modeling that demonstrates the preoperative anatomy, the surgical planning, and the postoperative anatomy. It also includes the preoperative echocardiography computed tomography scan images and the postoperative 4-dimensional flow magnetic resonance imaging image. Video available at: https://www.jtcvs.org/article/S2666-2507(24)00135-4/fulltext.
