# Supplementary figures and images for: A new approach to surgical correction of double-outlet right ventricle with remote interventricular communication
Source: JTCVS Tech. 2024 Mar 16;25:144–9. doi: 10.1016/j.xjtc.2024.03.006 (PMC11184593; doi:10.1016/j.xjtc.2024.03.006)

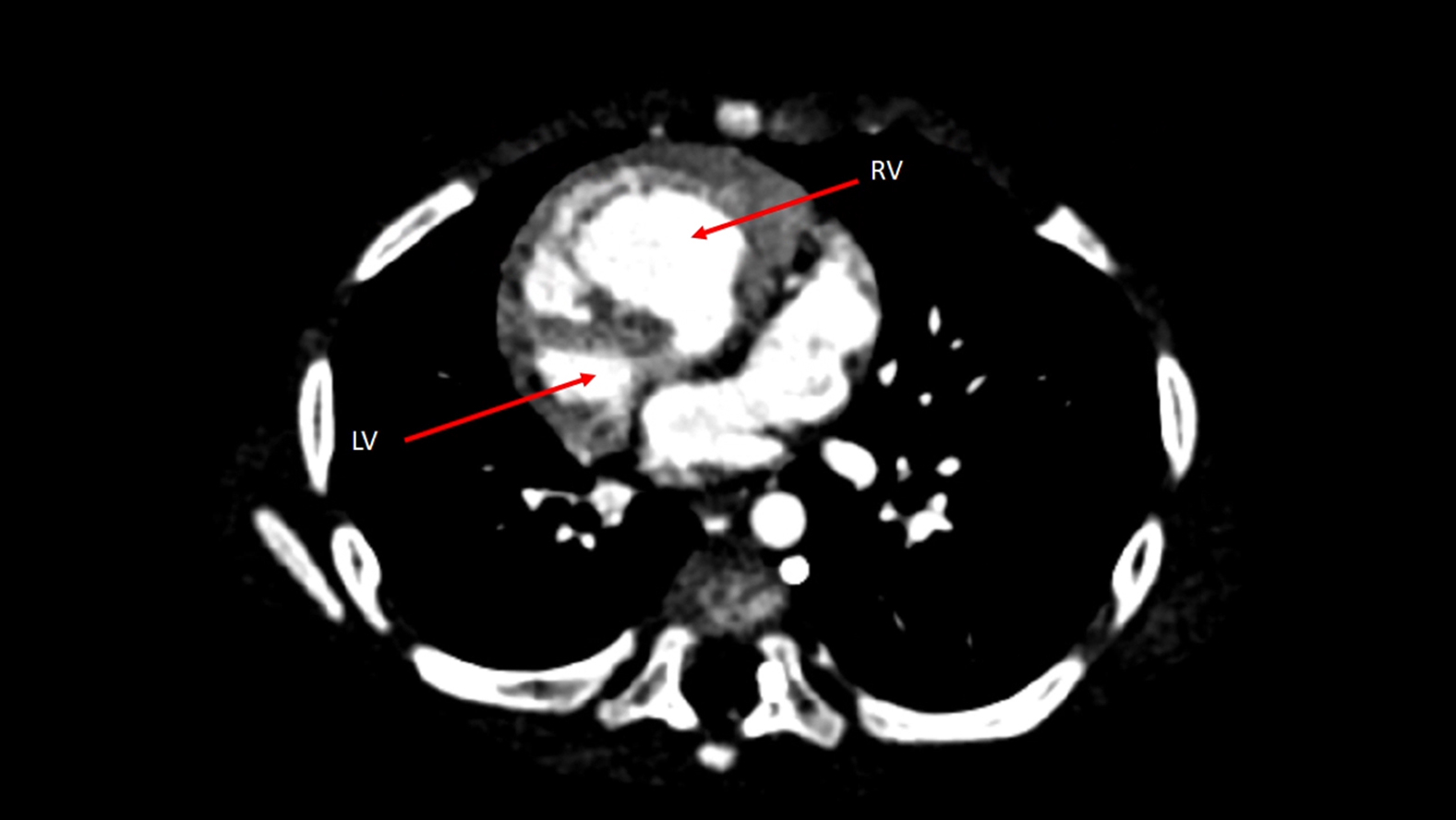

Supplement: Video 1 — The video clip, with its commentary, summarizes the case report and the 3-dimensional modeling that demonstrates the preoperative anatomy, the surgical planning, and the postoperative anatomy. It also includes the preoperative echocardiography computed tomography scan images and the postoperative 4-dimensional flow magnetic resonance imaging image. Video available at: https://www.jtcvs.org/article/S2666-2507(24)00135-4/fulltext. [file fx2.jpg]
